# X-ray microscopy reveals the outstanding craftsmanship of Siberian Iron Age textile dyers

**DOI:** 10.1038/s41598-021-84747-z

**Published:** 2021-03-04

**Authors:** Andreas Späth, Markus Meyer, Thomas Huthwelker, Camelia N. Borca, Karl Meßlinger, Manfred Bieber, Ludmilla L. Barkova, Rainer H. Fink

**Affiliations:** 1 Physical Chemistry II and Interdisciplinary Center for Molecular Materials, Friedrich Alexander University Erlangen-Nürnberg (FAU), Egerlandstraße 3, 91058 Erlangen, Germany; 2 Swiss Light Source (SLS), Paul Scherrer Institut, 5232 Villigen, Switzerland; 3 Physiology and Pathophysiology, Friedrich Alexander University Erlangen-Nürnberg (FAU), Universitätsstraße 17, 91054 Erlangen, Germany; 4 Ex Oriente, Waldleite 17, 97295 Waldbrunn, Germany; 5 Department of Eastern European and Siberian Archaeology, The State Hermitage Museum, 38 Dvortsovaya Embankment, 190000 Saint Petersburg, Russia; 6 Center for Nanoanalysis and Electron Microscopy (CENEM), Friedrich Alexander University Erlangen-Nürnberg (FAU), Egerlandstraße 3, 91058 Erlangen, Germany

**Keywords:** Scanning probe microscopy, Chemical physics, Imaging studies

## Abstract

The excellent craftsmanship of ancient Oriental and Central Asian textile dyers is already demonstrated in the remarkable brilliance and fastness of the colours of the so-called Pazyryk carpet, the by far oldest pile carpet found to date. This specimen resembles the advanced craftsmanship of Iron Age Central Asian textile production. We have employed synchrotron-based *µ*-XRF imaging to detect the distribution of metal organic pigments within individual fibres of the Pazyryk carpet (about 2500 years old) and compare the results to wool fibres, which we prepared according to traditional Anatolian dyeing recipes. We observe congruent pigment distribution within specimens from the Pazyryk carpet and natural wool fibres that we have fermented prior to dyeing. Therefore, we conclude that the superior fermentation technique has been utilized about 2000 years earlier than known so far.

## Introduction

Since centuries many people all over the world are fascinated by the brilliance and persistence of colours in traditional oriental carpets and flat weaves. Since natural dyes often consist of a mixture of various colourants, the resulting colours have a more vivid colour depth than synthetic dyes^1,2^. Many natural colourants require a mordant (typically a transition metal salt, e.g., KAlSO_4_, FeSO_4_, etc.) to form a metal–organic complex pigment^3–5^. Mordants typically increase the fastness of a dye by enhanced binding to the wool tissue, but can also influence the hue of the colour.

X-ray fluorescence microscopy (*µ*-XRF) is an ideally suited technique for high-resolution imaging of metal based pigments. Its outstanding capabilities in terms of spatial resolution and elemental sensitivity have been demonstrated for the analysis of pigments in cultural heritage samples, such as masterpiece paintings^6–8^, handwritings^9,10^, metal artefacts^11,12^ and other solid objects^13^. Here, we present the first study employing high-resolution *µ*-XRF for the in-situ analysis of pigments within a textile sample with high historical relevance. Furthermore, the specifics of the project (diameter of single wool fibres ~ 30–50 µm) required a spatial resolution excelling typical XRF studies on cultural heritage samples with lab-based X-ray sources by almost an order of magnitude^7,10^.

An outstanding example for the art of ancient natural dyeing is the Pazyryk carpet—a sheep wool pile carpet with a size of 1.83 × 2.0 m^2^ consisting of ~ 360,000 Turkish knots found in a Saka kurgan tomb from the 4th–3rd century B.C. in the Altai mountains (Fig. S1)^14,15^. The carpet was well preserved by a permafrost ice lens until its discovery by Russian archaeologists in 1947 and still shows vivid red and yellow colours. It is so far the oldest known pile carpet (dated to about 400 B.C.)—exceeding the next dated specimens by at least six centuries—and exhibits a mature craftsmanship that suggests several human generations of experience in production of such textiles^14^. Therefore, the Pazyryk carpet is a unique representative of ancient Central Asian cultural heritage.

Considering the excellent colour fastness it is of particular interest to understand the dyeing procedure used by the Pazyryk culture. Since the 1970s cultural anthropologists have studied and revived traditional Anatolian dyeing techniques based on fermentation of wool prior to dyeing^16,17^. They described a remarkable improvement of colour fastness against bleaching that is superior to textiles from industrial production for various natural colourants. Nevertheless, the fermentation technique had been almost forgotten at the time of these studies, because of its comparably slow production cycle (3 weeks for the fermentation procedure) and the risk of putrefaction when performed poorly^17^.

**Figure 1:**
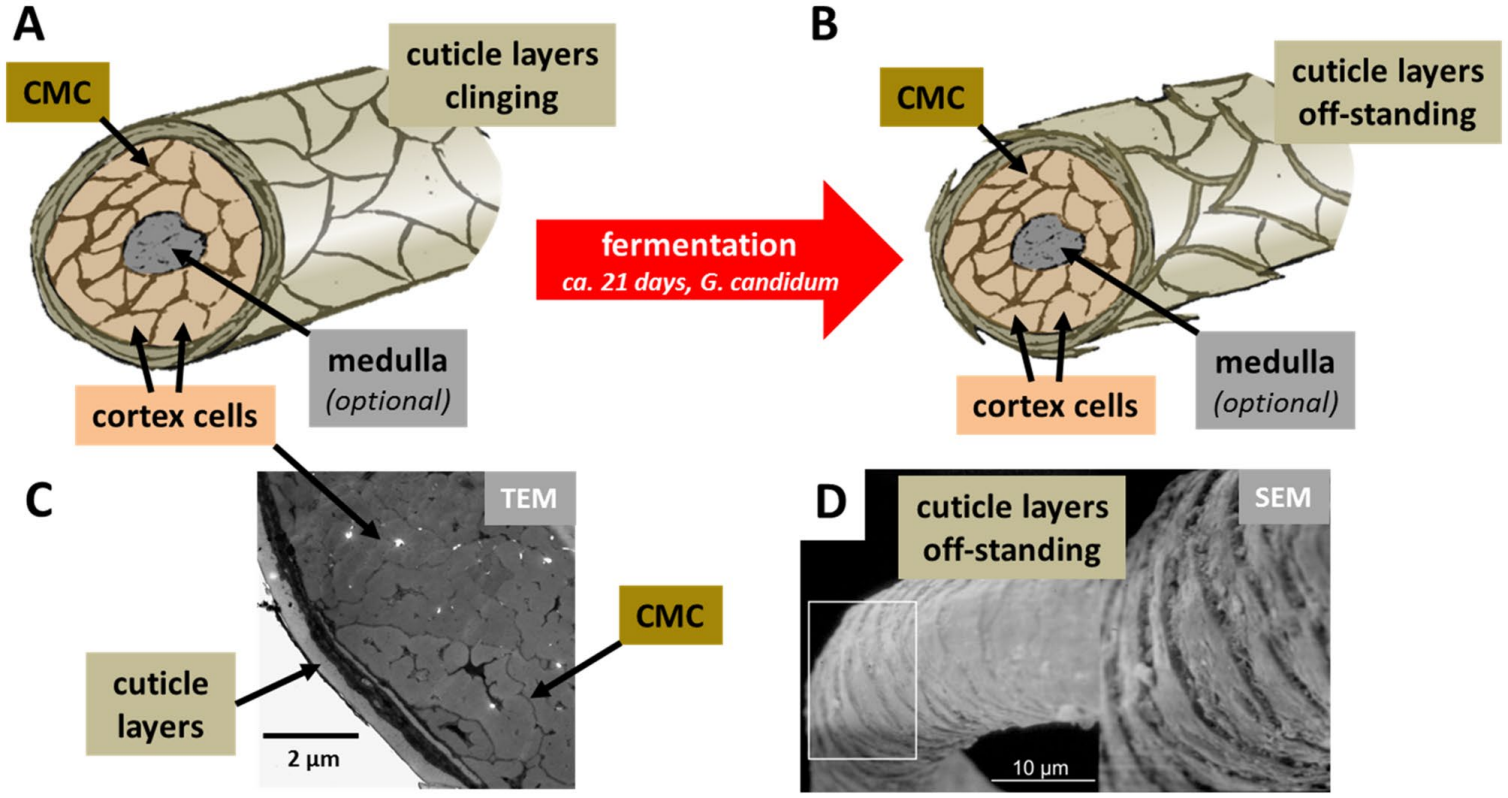
Scheme depicting the effects of fermentation on wool fibres. (**A**) Natural wool fibre depicting cortex cells, CMC and surrounding cuticle layers. The medulla is a porous structure inside thicker wool firers that is rarely found in Anatolian sheep shearings as used for carpet production. (**B**) After 3 weeks fermentation with *G. candidum*, the cuticle layers are protruding from the fibre. (**C**) TEM image depicting cortex, CMC and cuticle layers in a cross-section of Anatolian sheep wool. (**D**) SEM image of off-standing cuticle layers after fermentation.

Figure 1 depicts the morphological components of individual wool fibres and the influence of fermentation as confirmed by scanning electron microscopy (SEM)^18,19^. Fermentation with *G. candidum* yeast leads to an abduction of the outermost layers of the cuticle by consumption of the fatty interstices in-between the cuticle scales. Furthermore, *G. candidum* stabilizes the pH inside the hair to 4.4 resulting in saturation of naturally deprotonated thiol groups (R–S–H) within the cuticle. Both effects increase the permeability of the cuticle for metal–organic compounds^20,21^. Diffusion of metal–organic colourants inside the wool fibre takes places mainly inside the cell-membrane-complex (CMC)^20^. The CMC is a network of fatty acid layers that glues the keratin based components (cortex cells and cuticle scales) together^22^.

## Results and discussion

**Figure 2:**
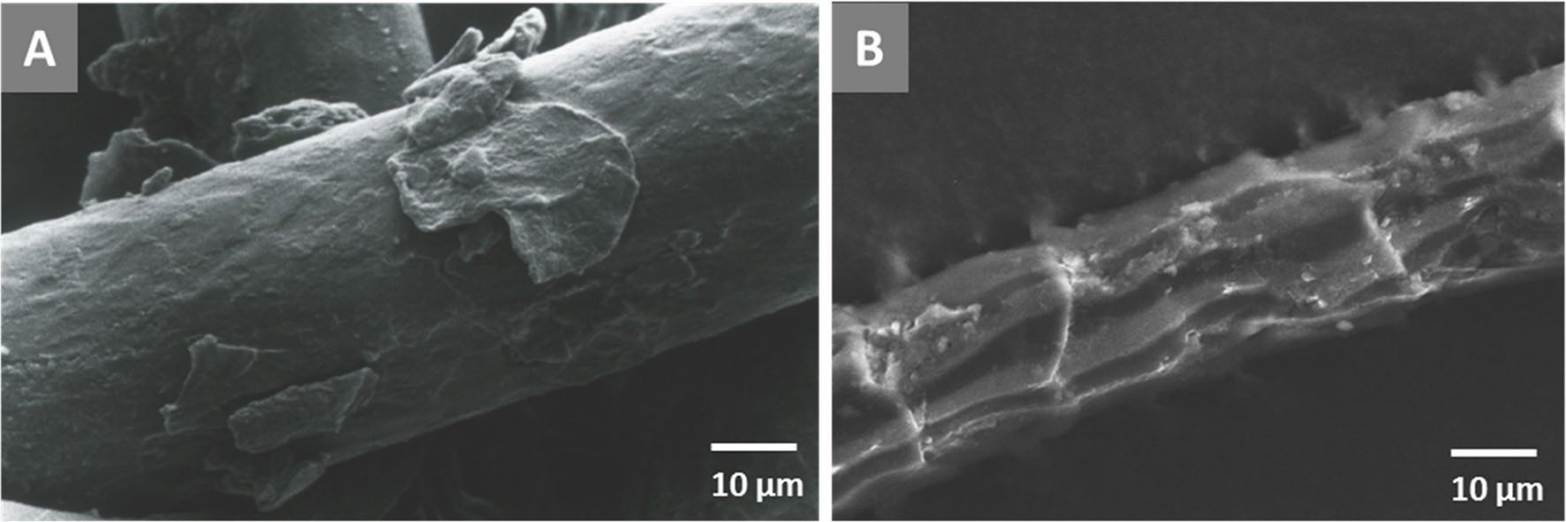
SEM micrographs of strongly abraded sheep wool fibres. (**A**) Wool fibre from a Turkish carpet from the eighteenth century. (**B**) Pazyryk carpet. The outermost cuticle scales are mainly lost by abrasion due to long-term use of the textile^17,19^. This finding is typical for ancient textiles unless they have been preserved very well. Therefore, the direct observation of scale abduction by fermentation of the wool prior to dyeing is usually not possible for ancient or intensely used specimens. The Pazyryk carpet fibres show significantly stronger surface abrasion than specimens from seventeenth or eighteenth century.

It is proposed that the increased colour fastness of fermented sheep wool stems from a deeper and also overall higher amount of uptake of pigments inside the fibres. In natural hair, the cuticle forms a moderate barrier between CMC and infiltrating metal–organic mordant dyes. After fermentation this diffusion channel is easier accessible and the dye is more likely to accumulate deep inside the fibre cortex causing a more intense and stable colouring. In transmission electron microscopy (TEM) images of cross-sections from fermented and dyed wool fibres the CMC appears darker than the surrounding cortex, which is not the case in not fermented, but identically dyed analogues (Fig. S2). We also see this effect in TEM micrographs of the Pazyryk carpet. However, this is no clear evidence of the presence of the pigment inside the CMC, since TEM does not provide sufficient chemical information. In principle SEM imaging can prove the fermentation process directly by detection of off-standing cuticle cells. However, this is usually only possible for recent or rarely used textiles, since long-term use leads to a loss of the outer cuticle layers by abrasion (Fig. 2A)^17^. Figure 2B shows an SEM image of an exemplary fibre from the Pazyryk carpet. The surface of these fibres is heavily damaged and no direct conclusions from the shape of cuticle layers can be deduced. At some positions the cuticle layers are completely missing. Therefore, the study of the dyeing procedure applied to the Pazyryk wool requires a high resolution detection of pigments within the fibres.

We have prepared freshly sheared wool from Anatolian sheep according to traditional dyeing procedures as passed on in Eastern Anatolia since generations^16,17^. All specimens were dyed with madder (*R. tinctorum*) and KAl(SO_4_)_2_, because the resulting pigment Turkey red is one of the most common red natural colourants in Middle East since centuries^2^. The use of *R. tictorum* by the Pazyryk culture has been shown by chromatography and UV/Vis spectroscopy^23^ of extracted pigment. The fibres were embedded in epoxy and cross-sections were prepared by microtome sectioning. We have also used scanning transmission X-ray microscopy (STXM) to verify that the morphology (also in terms of chemical composition) of the ancient fibres were not altered by age, which might have caused an a posteriori migration of pigments within the fibres (cf. Fig. S3).

**Figure 3:**
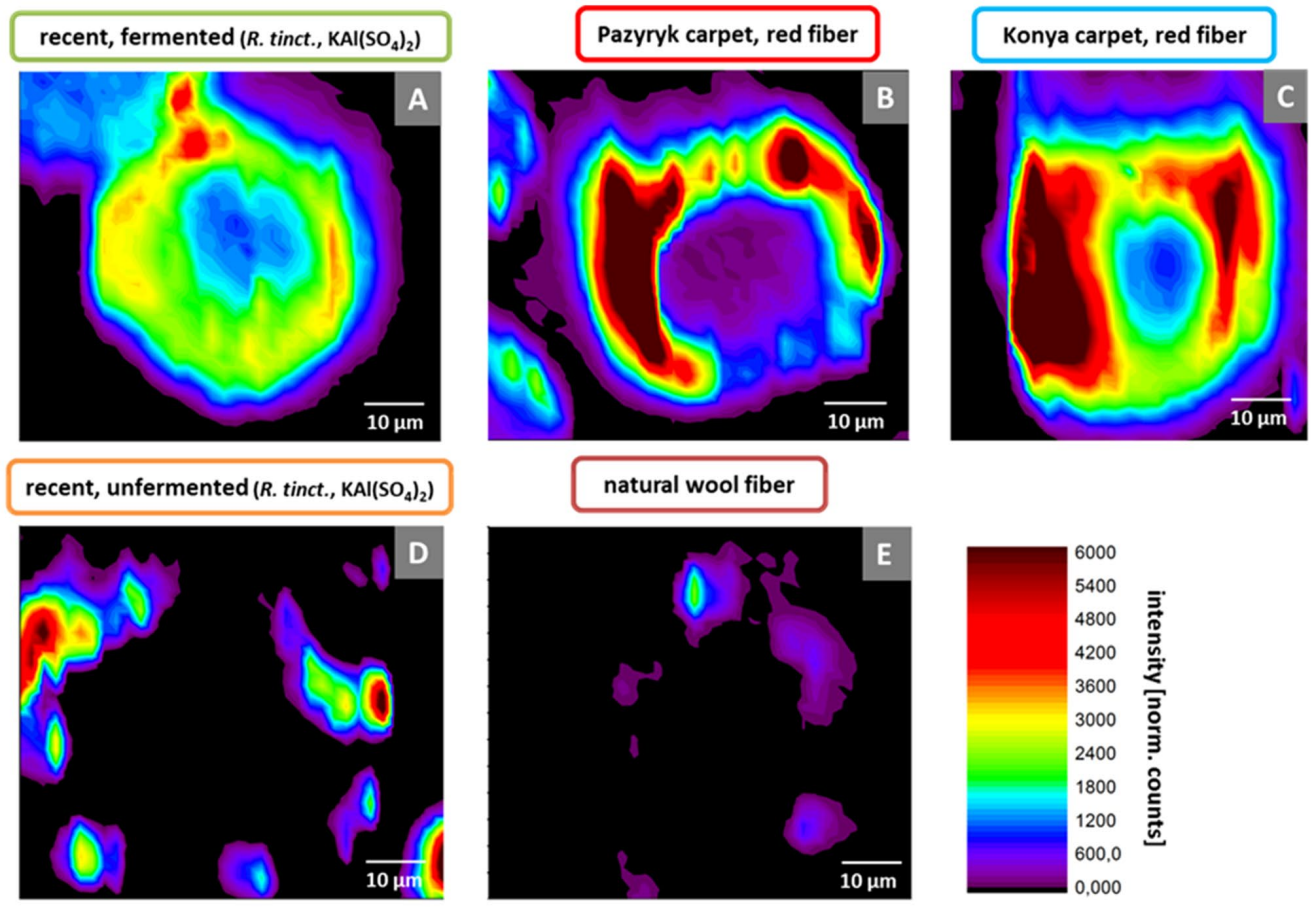
*µ*-XRF maps of various sheep wool specimens at the Al *K*-edge. All maps have been recorded with a pixel size of 2 × 2 µm^2^. (**A**) Recently fermented and dyed with *R. tinctorum*/KAl(SO_4_)_2_. (**B**) Red fibre from Pazyryk carpet. (**C**) Red fibre from a carpet from the eighteenth century (origin: Konya, Turkey). (**D**) Recently dyed with *R. tinctorum*/KAl(SO_4_)_2_, no fermentation. (**E**) Natural wool fibre. The maps provide an in-situ visualization of the pigment distribution within the wool fibres. They show an increased uptake of Al especially into the inner regions of the wool fibres when fermentation has been applied prior to dyeing.

Figure 3 shows high-resolution *µ*-XRF maps of several individual wool fibres from various specimens. The aluminium distribution along the cross-section of the recently dyed fibres is compared to samples from the Pazyryk carpet and an eighteenth century carpet from Turkey as well as natural wool fibres from the same batch as the recently dyed specimens. For the recently fermented wool (Fig. 3A) we detect a deep penetration of a significant amount of aluminium into the fibre cortex with a strong gradient from the cuticle towards the centre of the fibre, while the not fermented analogue (Fig. 3D) shows aluminium just within the outer part of the cuticle, although the dyeing procedure was the same in both cases. The undyed natural wool fibre (Fig. 3E) shows only minor contamination on the surface of the fibre as to expect for a sample from a natural source. The Pazyryk carpet and the Konya carpet show very similar characteristics in their aluminium distribution with a gradient from the cuticle towards the fibre centre, but still a significant amount of aluminium deep inside the cortex. Based on the fluorescence maps of the investigated specimens we have also plotted radial profiles of the respective aluminium distribution to visualize the corresponding characteristics of recently fermented and Pazyryk fibres (Fig. S4). For S *K*-edge maps of the wool fibres themselves, cf. Fig. S5 (S *K*-edge maps).

**Figure 4:**
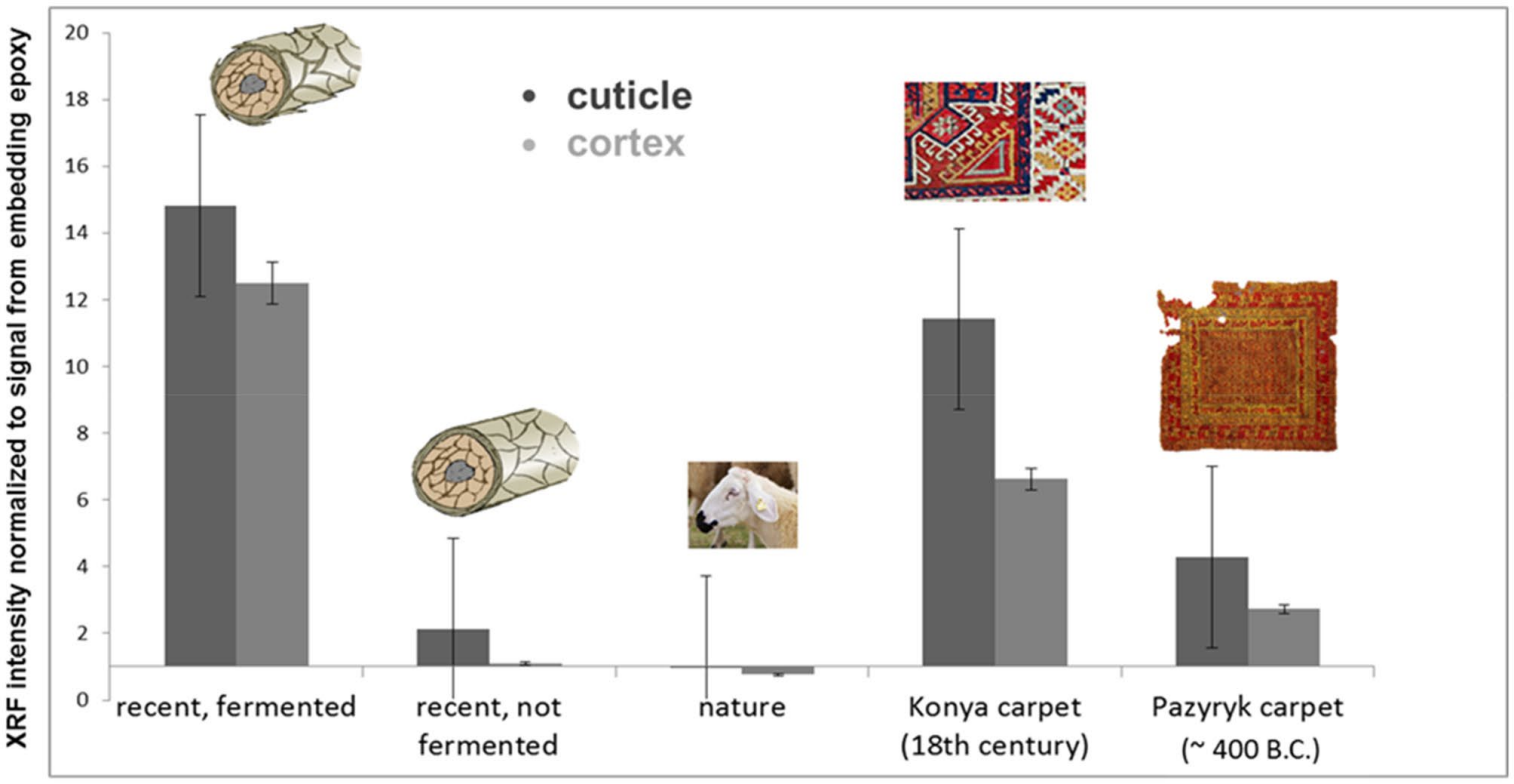
Normalized Al *K*-edge intensities equivalent to pigment uptake for various wool specimens with respect to depth of penetration. Each column represents an averaged value derived from *µ*-XRF maps of 5–8 individual fibres. Normalization was conducted with respect to the embedding epoxy. Therefore, it was possible to correct for geometric effects from varying sample dimensions (cf. Fig. S6). A strong enhancement of the Al concentration especially in the cortex of the fibres of the Konya and Pazyryk carpet wool is attributed to fermentation prior to dyeing. Averaging over several fibres of each batch minimizes variances from asymmetric embedding or local density variations within individual fibres that are given by nature. Error bars reflect standard deviation.

We recorded *µ*-XRF maps of several individual wool fibres for all specimens. The averaged and normalized fluorescence intensities are depicted in Fig. 4. In all cases we compare also the outer part of the fibre (mainly cuticle) with the inner part (mainly cortex). While the natural wool sample shows almost no aluminium (in the cuticle it is even less than in the embedding epoxy used as reference for normalization), the not fermented, but dyed fibres took up the pigment to some extend into the cuticle, but not into the cortex. All other samples show a gradient of aluminium towards the fibre centre. However, the overall amount of pigment is much higher in the fermented sample and moreover, a significant uptake within the inner cortex is detected. The variations in the absolute amounts of aluminium uptake for the recently fermented and the ancient specimens might be caused by leaching, but of course the initial concentrations of the dyeing solutions are unknown for the Konya and Pazyryk fibres.

We conclude from our studies that both the eighteenth century Konya carpet and the Pazyryk carpet have been manufactured from wool that was fermented prior to dyeing. This means that the people of the Pazyryk culture not only already had sophisticated knowledge about pile carpets, but were also highly skilful textile dyers achieving colour fastness superior to modern industrial production. Our results also proof that the fermentation technique was in ancient times not only restricted to Eastern Anatolia and may have played an important role in traditional dyeing craftsmanship. Furthermore, we have demonstrated the high potential of *µ*-XRF imaging in analysis of textile cultural heritage and see great potential for further high-resolution in-situ studies in this field.

## Materials and methods

### Sample preparation

The recently prepared wool was obtained from Anatolian weavers and stems from traditional Anatolian fat-tailed sheep (*Ak Karaman*). The authors did not handle the animals themselves. The process of dyeing for the recently prepared wool fibres is described in more detail in^17^. The fermentation process is started with a suspension of sourdough and wheat bran. The latter fosters selective growth of *G. candidum* yeast due to a high content of pentosan. The *G. candidum* culture regulates the pH to 4.4 and hinders the growth of putrefactive bacteria. Within about 3 weeks of fermentation *G. candidum* decomposes lipids inside the cuticle layers of the wool enhancing permeability within the subsequent dyeing process. After fermentation madder roots and mordant are added to the suspension at ambient conditions. Red fibres are obtained with KAl(SO_4_)_2_ mordant (20 vol.-% solution)^19^. The unfermented specimens have been dyed under identical conditions without previous fermentation.

The specimen from the Pazyryk carpet has been extracted with permission from The State Hermitage Museum, St. Petersburg, from a loose part of the carpet designated for scientific studies. The specimen from the Konya carpet has been extracted with permission from Museum for Islamic Art, Berlin, at an inconspicuous position. Both specimens have been kept under clean and dark conditions until embedding.

The various specimens were embedded in an epoxy resin derived from a 1:1 mixture of 4,4′-Methylenebis(2-methylcyclohexylamine) and Trimethylolpropane triglycidyl ether. This epoxy provides very good contrast in X-ray microscopy of biological samples at the C *K*-edge^24^. After drying for 3 days at room temperature, the sample blocks were trimmed to pyramidal shape and microtomed with a diamond knife to obtain a flat surface along the cross section of several isolated wool fibres (Fig. S6) for *µ*-XRF mapping. This sample preparation procedure was used, since previous *µ*-XRF mapping on thin sections of various thicknesses (200 nm–3 µm) was not successful. The overall amount of Al within the corresponding small sample volumes of these thin sections was below the detection limit of the method.

Thin sections (~ 200 nm) gained during microtoming of the sample blocks were transferred to TEM grids and used for STXM imaging.

Samples for TEM imaging have been embedded in EpoFix (Struers GmbH). After drying over night at room temperature the hardened material was trimmed and microtomed to ~ 100 nm thin cross sections.

### Electron microscopy

TEM imaging was performed with a Zeiss LEO 912 Omega operated at 80 kV and in low magnification mode. SEM imaging was performed with a Zeiss Evo 40 (both instruments at Physical Chemistry II, FAU). The SEM is also equipped with an EDX detector. However, EDX mapping did not provide sufficient detection sensitivity to achieve useful results on the Al distribution within the investigated specimens.

### STXM imaging

STXM measurements where performed at the PolLux beamline at the Swiss Light Source in Villigen, Switzerland^25^. The micrographs were recorded at the C *K*-edge resonance of keratin (288.1 eV)^24^ with a 25 nm Ni Fresnel zone plate, a pixel size of 25 × 25 nm^2^ and a dwell time of 10 ms per pixel.

### *µ*-XRF imaging

*µ*-XRF studies were performed at the PHOENIX beamline at the Swiss Light Source. The X-ray beam was focused to 3 × 5 µm^2^ and the pixel size for each recording was 2 × 2 µm^2^. The size of the focal spot was determined with a beam profiler system based on a CCD camera and a fluorescent Ce doped YAG crystal. The excitation energy for fluorescence mapping was 2.05 keV and the dwell per pixel was 2 s. The samples were mounted under 45° degree relative to the incoming beam to allow penetration of X-rays into the sample and exit of elemental fluorescence signals from the specimen. Fluorescence maps were recorded by scanning of the sample relative to the fixed position of the microbeam. The energy dispersive X-ray fluorescence spectra were recorded for each position using a four-element solid state detector (Vortex, Hitachi U.S.A.) with 160 eV energy resolution. The detector has four silicon drift sensors covered by an AP3-type window, consisting of an ultrathin polymer film supported by a Si grid structure (open area = 77%). The elemental X-ray transmission for Al Kα radiation is 75%. For the best quality of the Al *K*_*α*_ emission line in the fluorescence spectra in terms of signal to noise ratio and intensity, the excitation energy was chosen at 2.05 keV, as close as possible to the Al *K* edge. However, at this photon energy the sulfur emission line (for proper fibre localization) cannot be recorded, as the S *K*-edge (2.47 keV^26^) is above the energy of the incoming photons. Therefore, the higher order suppression of the beamline was deactivated, resulting in a small portion of third order light from the monochromator (6.15 keV). We calculated the contribution of the third order light by measuring a KAl(SO_4_)_2_ standard (3.0 mg in 100 mg cellulose). This allowed calculating the portion of higher order light to be ~ 2%, while 98% was first order light. This low portion was sufficient to record S *K*-edge maps, but will not significantly affect the Al *K*-edge maps.

### Data evaluation

STXM maps have been analyzed with aXis2000.

Fluorescence maps have been fitted and analyzed with PyMca^27^. An exemplary fit is depicted in Fig. S7. The attenuation of the excitation and fluorescence photons within the hair fibres was calculated based on literature values for the mean elemental distribution of chemical elements in sheep wool^22,28^, the mean density of wool fibres (1.4 g/cm^3^)^22^ and respective attenuation factors^26^. With this method we could calculate the energy dependent probing depth (~ 20 µm at 2.05 keV, ~ 360 µm at 6.15 keV) and correlate the fluorescence signals to the qualitative metal contents. An exact quantitative evaluation of the Al content within the specimens would not be legitimate, since the density of the wool fibres is by nature not exactly determined. Therefore, we restricted our analysis to qualitative measures that are reliable for each individual fibre. We have estimated that the mean Al content within the cuticle of fermented fibres (highest measured intensities) is in the order of 0.1%.

For comparison of the Al *K*-edge fluorescence intensities from different specimens, the respective values have been normalized by division to the Al signal from the embedding epoxy. The epoxy should have a constant distribution of Al traces (confirmed by constant intensity in Al *K* edge emission). This method allows for a proper treatment of geometrical variances of the epoxy stubs leading to slightly varying incidence angle for each sample.

## Supplementary Information

Supplementary Information.

## Data Availability

All data needed to evaluate the conclusions in the paper are present in the paper and the Supplementary Materials. Additional data related to this paper may be requested from the authors.
