# Supplementary material for: X-ray microscopy reveals the outstanding craftsmanship of Siberian Iron Age textile dyers
Source: Sci Rep. 2021 Mar 4;11:5141. doi: 10.1038/s41598-021-84747-z (PMC7970846; doi:10.1038/s41598-021-84747-z)
Supplement: Supplementary file 1 — Supplementary Information. [file 41598_2021_84747_MOESM1_ESM.pdf]

Supplementary Information

for

X-ray microscopy reveals the outstanding craftsmanship of Siberian Iron Age textile dyers

Andreas Späth[1,*,+], Markus Meyer[1,+], Thomas Huthwelker[2], Camelia N. Borca[2], Karl Meßlinger[3], Manfred Bieber[4], Ludmilla L. Barkova[5,†], Rainer H. Fink[1,6,*]

[1] Physical Chemistry II and Interdisciplinary Center for Molecular Materials, Friedrich Alexander University Erlangen-Nürnberg (FAU), Egerlandstraße 3, 91058 Erlangen, Germany.

[2] Swiss Light Source (SLS), Paul Scherrer Institut, 5232 Villigen, Switzerland.

[3] Physiology and Pathophysiology, Friedrich Alexander University Erlangen-Nürnberg (FAU), Universitätsstraße 17, 91054 Erlangen, Germany.

[4] Ex Oriente, Waldleite 17, 97295 Waldbrunn, Germany.

[5] Department of Eastern European and Siberian Archaeology, The State Hermitage Museum, 38 Dvortsovaya Embankment, 190000 Saint Petersburg, Russia.

[6] Center for Nanoanalysis and Electron Microscopy (CENEM), Friedrich Alexander University Erlangen-Nürnberg (FAU), Egerlandstraße 3, 91058 Erlangen, Germany.

* andreas.spaeth@fau.de; rainer.fink@fau.de

+ these authors contributed equally to this work

† deceased

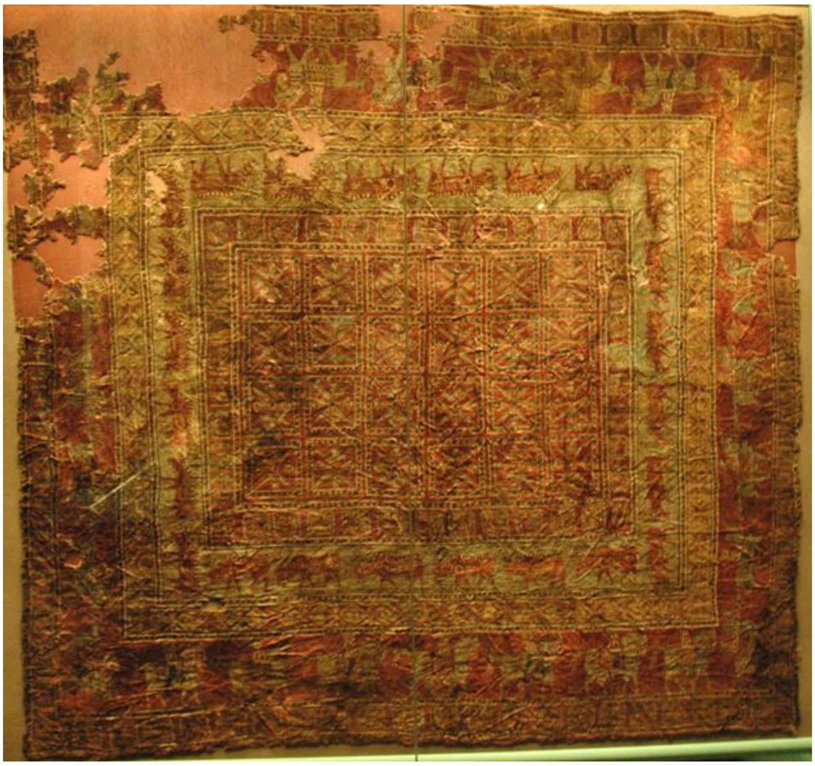

Figure S1.

Photograph of the Pazyryk carpet in the exhibition of The State Hermitage Museum, St. Petersburg. The fibres for the present analysis have been extracted from a loose piece of the carpet belonging to a missing part in the upper left edge of the photograph that is stored by the museum for scientific purposes. The carpet has an overall size of 1.83 x 2.0 m^2.

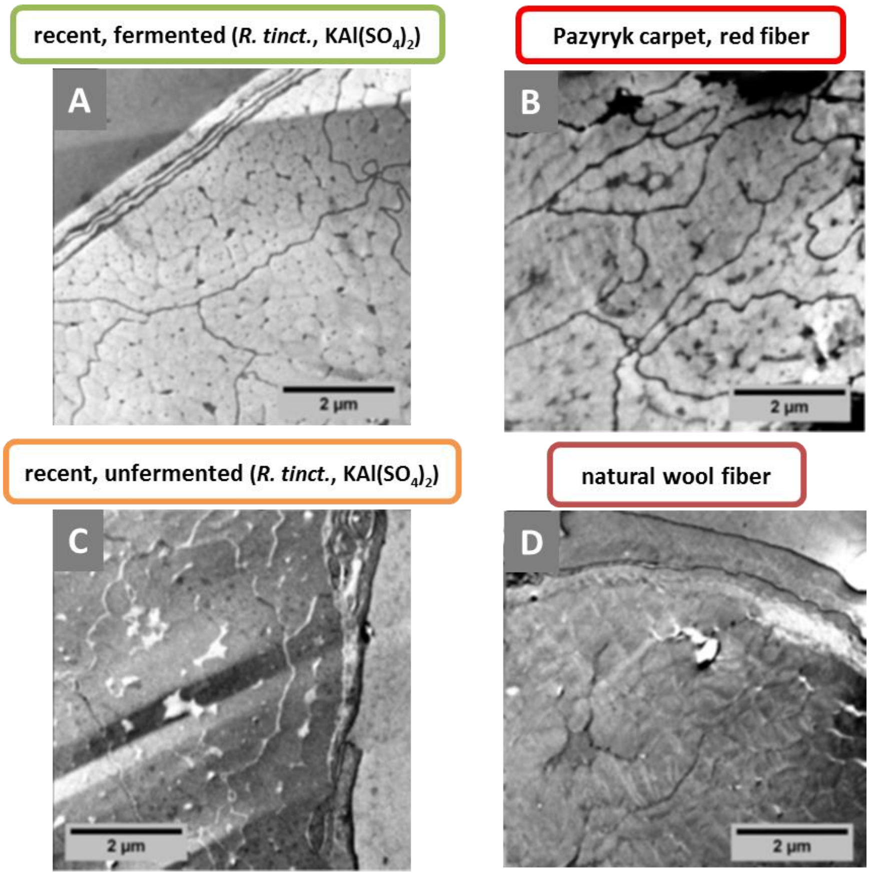

Figure S2.

TEM micrographs of sheep wool. A) Recently fermented and dyed with madder roots and $KAl(SO_4)_2$. B) Pazyryk carpet. C) Recently dyed with madder roots and $KAl(SO_4)_2$, no fermentation. D) Natural wool. A) and B) show a darker contrast for the CMC compared to the cortex, while the same structure appears brighter in C). In B), however, the CMC has poor contrast to the cortex. Since diffusion inside the wool fibres is mainly happening within the CMC, these differences in the contrast may be a hint on various amounts of pigment uptake or morphological changes during the fermentation process. However, without further chemical information as provided by μ-XRF imaging, this interpretation stays speculative.

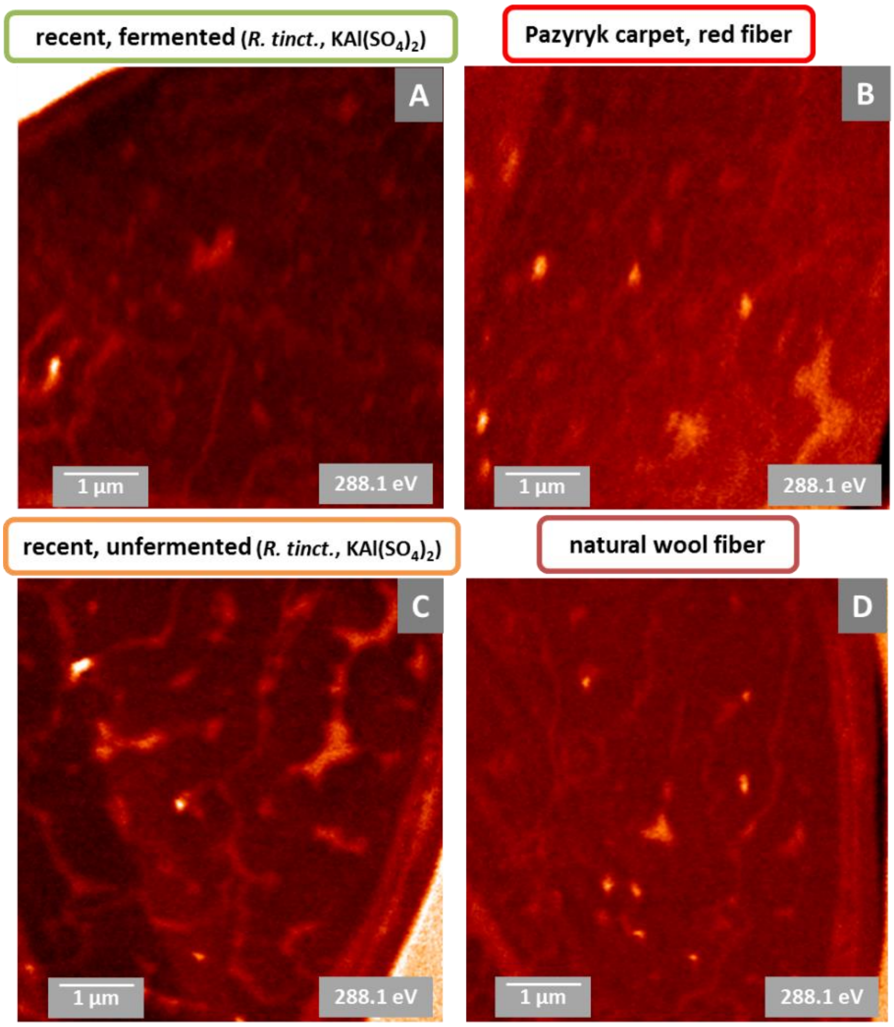

Figure S3.

STXM micrographs at C *K*-edge resonance of keratin. The applied incident photon energy (288.1 eV) resulted optimum contrast for keratinous material (cuticle, cortex) vs. CMC[25]. A) Recently fermented and dyed with madder roots and $KAl(SO_4)_2$. B) Pazyryk carpet. C) Recently dyed with madder roots and $KAl(SO_4)_2$, no fermentation. D) Natural wool. The STXM images confirm that the morphology of the wool fibres is still intact after fermentation and that this is also true for the Pazyryk fibres despite their long-term burial. STXM is in that sense more sensitive to chemical composition than TEM.

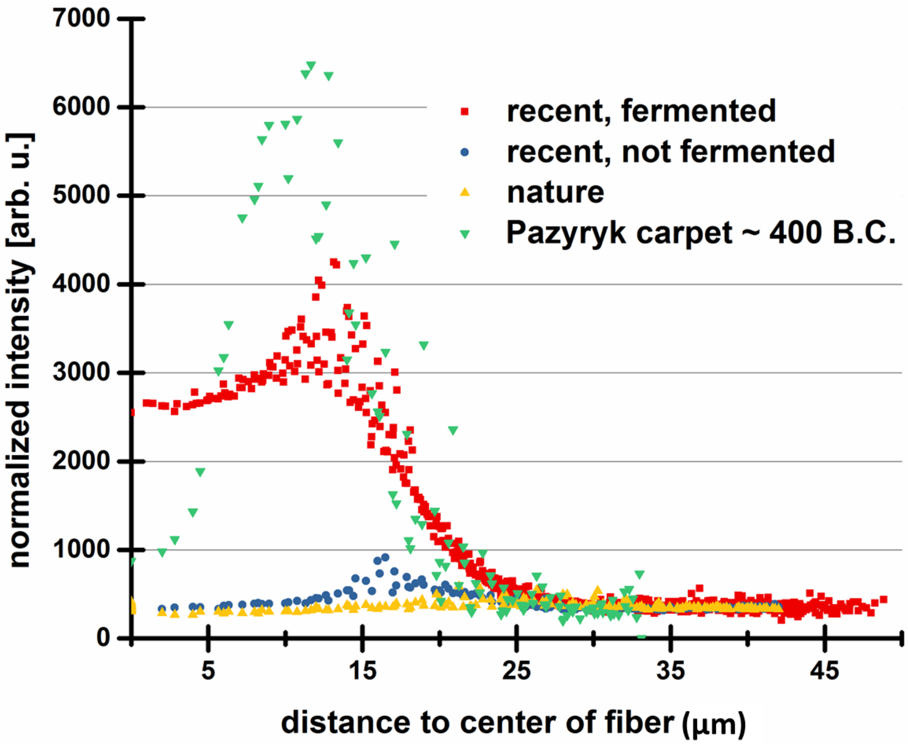

Figure S4.

Radial profiles of aluminium distribution derived from *µ*-XRF maps in Fig. 2. The respective radial profiles corroborate the consideration that recently prepared fermented wool and fibres from the Pazyryk carpet have similar characteristics in their respective aluminium distribution, while not fermented wool has a significantly lower overall Al content that is only enriched within the outermost layers of the fibres. (Konya carpet (Fig. 3C) is omitted for clarity).

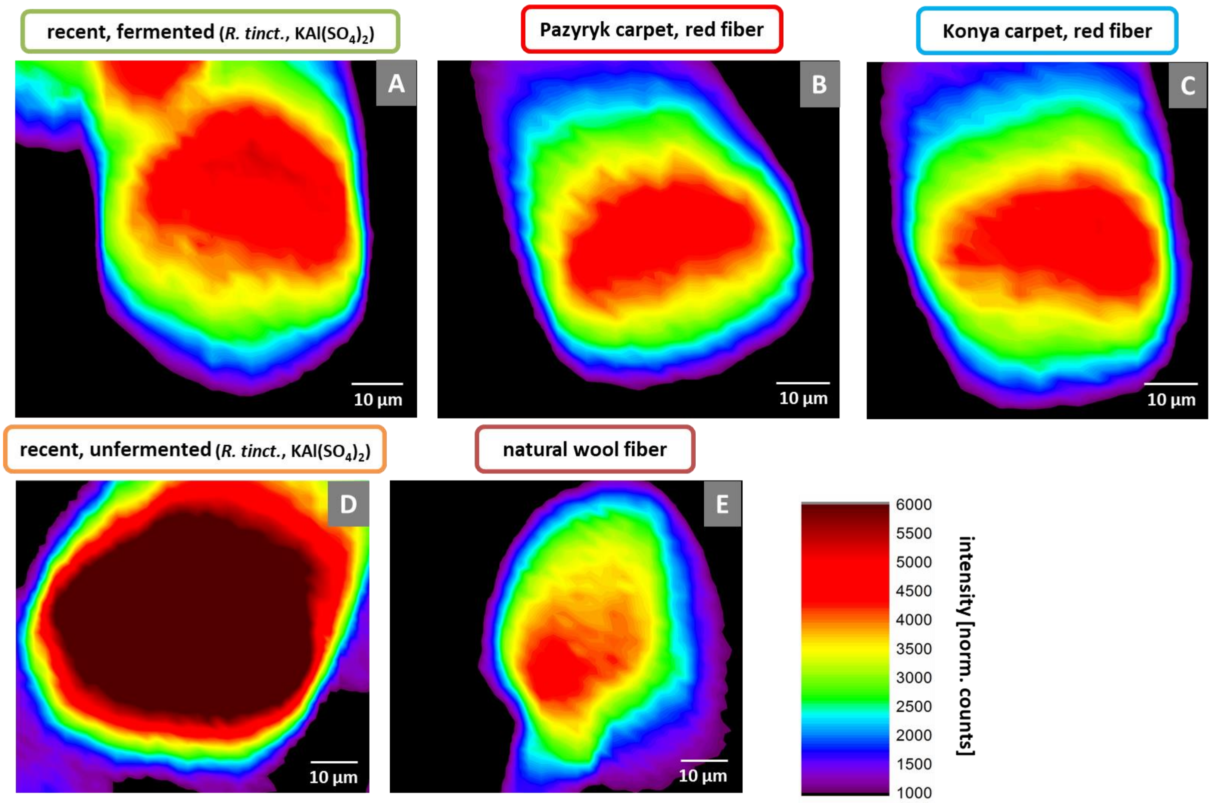

Figure S5.

***µ*-XRF maps of various sheep wool specimens at the S *K*-edge.** All maps have been recorded with a pixel size of 2x2 µm². A) Recently fermented and dyed with *R. tinctorum* / $KAl(SO_4)_2$. B) Red fibre from Pazyryk carpet. C) Red fibre from a carpet from the 18th century (origin: Konya, Turkey). D) Recently dyed with *R. tinctorum* / $KAl(SO_4)_2$, no fermentation. E) Natural wool fibre. The S maps help to define the position the wool fibres, especially for specimens with overall low aluminium uptake. Due to a higher penetration depth, the fibres might, however, appear more asymmetric, since we see more of the continuation of the fibres within the epoxy block and the fibres have a low probability to be embedded straight in-line with the surface normal of the prepared cross section.

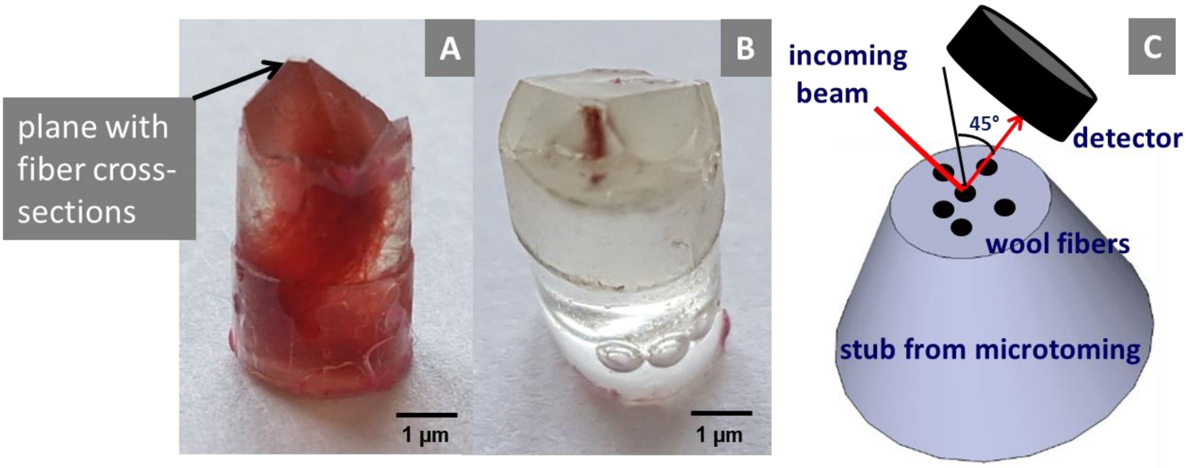

Figure S6.

Preparation of samples for *µ*-XRF mapping. A) Recently dyed and fermented wool in epoxy. B) Pazyryk sample. C) Instead of measuring thin sections, the microtome was used to prepare proper cross sections of the wool fibres within the epoxy stub. These stubs were placed in the focus of the X-ray beam under 45° according to the optical axis of the beam as well as the detector.

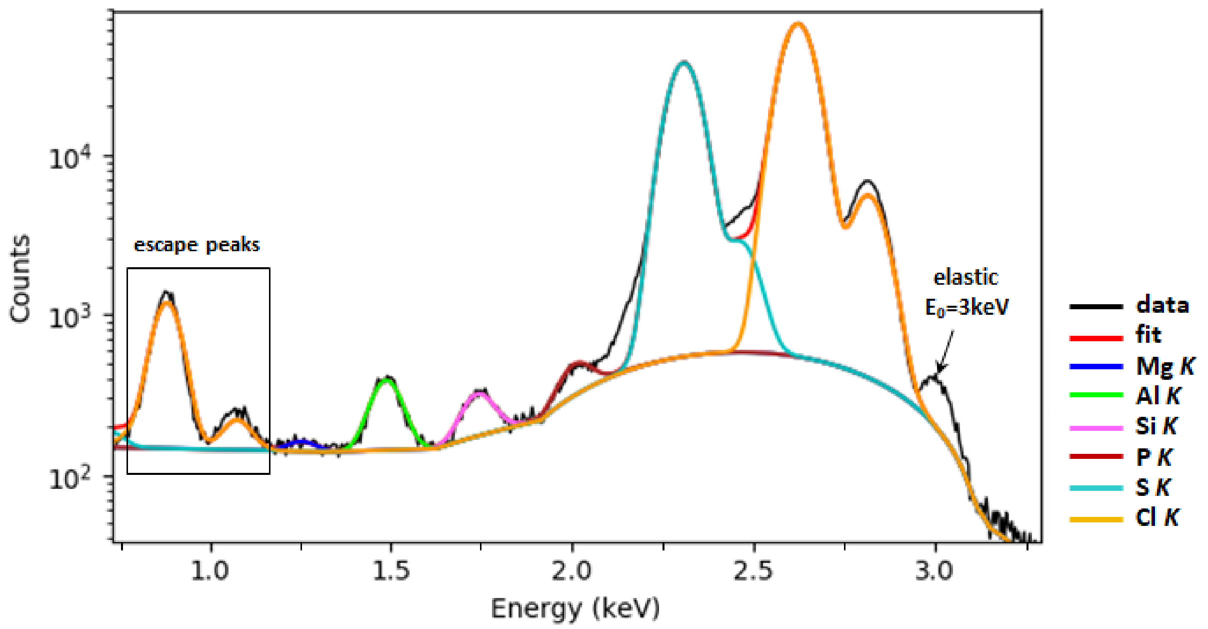

Figure S7.

Fit for the fluorescence emission spectrum derived by summation over an exemplary specimen within a 2D µ-XRF map. The high contribution of Cl stems from the embedding epoxy. The depicted peak fitting is the basis for the setting of valid photon energy borders during visualization of 2D elemental distribution maps (e.g., Al or S *K*-edge).
